# Investigation of Clinical Characteristics and Etiological Factors in Children with Molar Incisor Hypomineralization

**DOI:** 10.1155/2018/7584736

**Published:** 2018-05-09

**Authors:** Maria Rita Giuca, Maria Cappè, Elisabetta Carli, Lisa Lardani, Marco Pasini

**Affiliations:** Department of Surgical, Medical, Molecular Pathology and Critical Area, Dental and Oral Surgery Clinic, Unit of Pediatric Dentistry, University of Pisa, Via Savi 10, 56126 Pisa, Italy

## Abstract

**Aim:**

The purpose of the present study was to evaluate the clinical defects and etiological factors potentially involved in the onset of MIH in a pediatric sample.

**Methods:**

120 children, selected from the university dental clinic, were included: 60 children (25 boys and 35 girls; average age: 9.8 ± 1.8 years) with MIH formed the test group and 60 children (27 boys and 33 girls; average age: 10.1 ± 2 years) without MIH constituted the control group. Distribution and severity of MIH defects were evaluated, and a questionnaire was used to investigate the etiological variables; chi-square, univariate, and multivariate statistical tests were performed (significance level set at *p* < 0.05).

**Results:**

A total of 186 molars and 98 incisors exhibited MIH defects: 55 molars and 75 incisors showed mild defects, 91 molars and 20 incisors had moderate lesions, and 40 molars and 3 incisors showed severe lesions. Univariate and multivariate statistical analysis showed a significant association (*p* < 0.05) between MIH and ear, nose, and throat (ENT) disorders and the antibiotics used during pregnancy (0.019).

**Conclusions:**

Moderate defects were more frequent in the molars, while mild lesions were more frequent in the incisors. Antibiotics used during pregnancy and ENT may be directly involved in the etiology of MIH in children.

## 1. Background

The term “molar incisor hypomineralization (MIH)” is a definition introduced by Weerheijm et al. to describe enamel defects affecting the first permanent molars and, frequently, permanent incisors; furthermore, the second permanent molars and permanent canines can also be involved [1].

MIH is a relatively common condition with a world prevalence range from 2.8% to 44% [2]. Mulic et al. observed that females exhibit a higher prevalence of mineralization than males of the same age and that the maxillary first molars and incisors were more often affected in comparison to mandibular teeth [3].

Clinical features of MIH include white-to-yellow/brown large demarcated porous opacities caused by changes in mineral and protein enamel composition, anomaly in the tissue translucency, tooth hypersensitivity that is due to the exposure of dentin, posteruption enamel breakdown, and rapid dental caries progression.

In a systematic review of Americano et al., a positive association between dental caries and MIH was found as the enamel breakdown predisposes for higher dental biofilm accumulation. The authors concluded that children with MIH were 2 to 4 times more likely to show caries than young patients of the control group [4].

Bozal et al. [5] evaluated the ultrastructural aspects of the surface of the teeth with MIH and found a loss of prismatic pattern, a porous ultrastructure with cracks, decreased levels of calcium and phosphate, and alterations in ionic composition. These enamel alterations may interfere with the dental restorative procedures.

**Figure 1:**
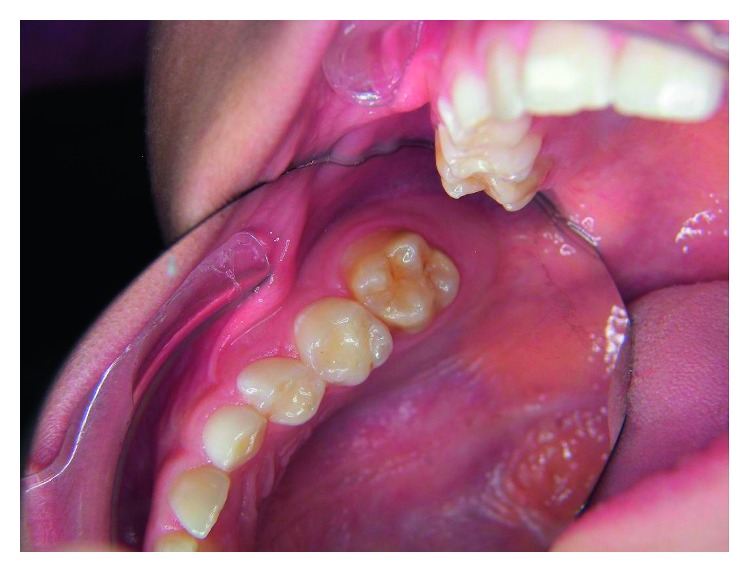
Mild molar hypomineralization.

**Figure 2:**
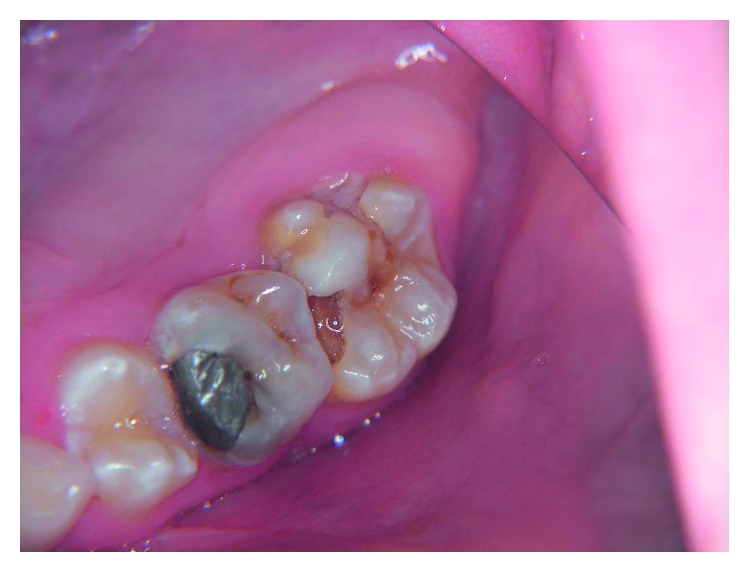
Severe molar hypomineralization.

**Figure 3:**
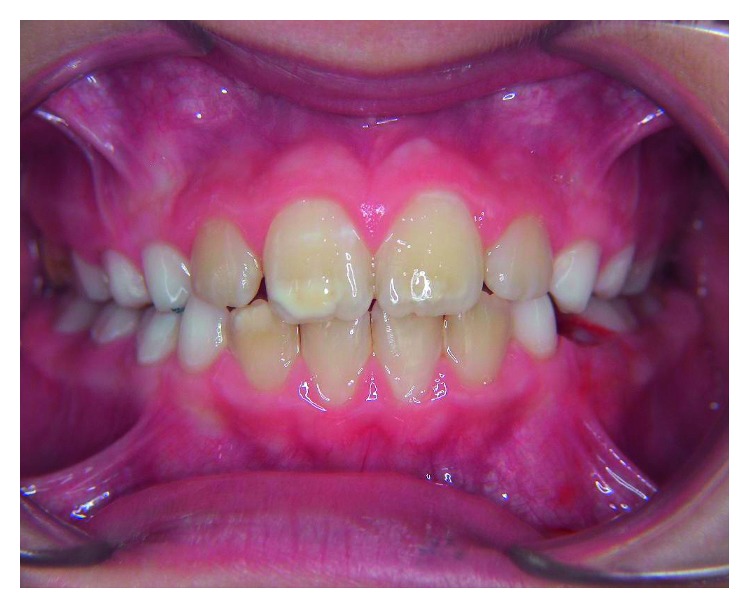
Mild incisor hypomineralization.

**Figure 4:**
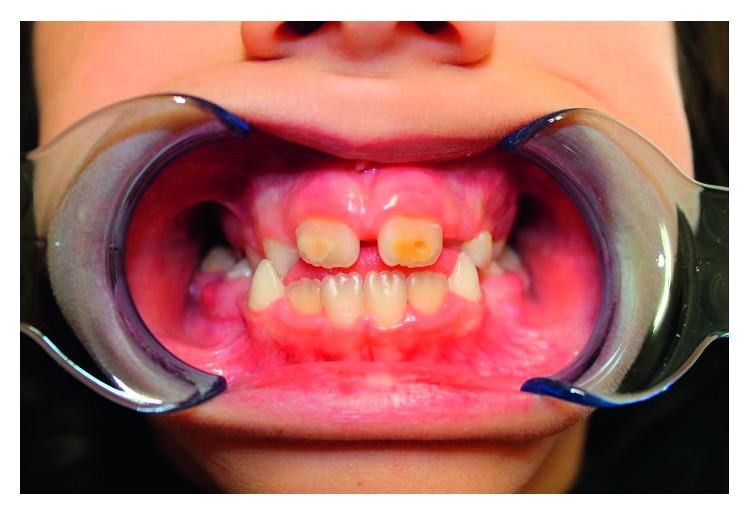
Severe incisor hypomineralization.

MIH lesions can be classified into three categories: mild (isolated enamel opacities without enamel sensitivity), moderate (occlusal or incisal third involvement with no or slight sensitivity), and severe (presence of posteruptive enamel breakdown and widespread caries that determine both functional and esthetic complications) (Figures 1–4) [6].

Early detection, intervention, and appropriate therapy can prevent severe complications and improve both masticatory function and esthetic. It was stated that the age of 8 years is the best age for a correct diagnosis, as at this stage, all upper and lower permanent incisors and mandibular and maxillary permanent first molars are fully erupted [7]. Differential diagnosis includes amelogenesis imperfecta, hypoplasia, and fluorosis.

Although the etiology of MIH is still not clear, a combination of different factors that may affect the ameloblasts during the enamel formation has been proposed. There is not often a family history of enamel hypomineralization such as in cases of amelogenesis imperfecta.

Mineralization of the first permanent molars usually starts at birth (just before or shortly after birth), and it is fully completed at 4-5 years of age [8]; anomalies that occur during the enamel matrix secretion cause enamel hypoplasia, while enamel anomalies during the maturation stage can determine the onset of hypomineralization.

In a recent systematic review [9], a possible link between MIH lesions and both systemic and environmental factors that may play a role during the enamel maturation stage was suggested.

Health problems that occurred during pregnancy and early childhood illness (i.e., asthma and pneumonia) were selected as the main possible etiological factors.

However, as the authors stated, the validity of several previous reports was impaired by poor protocol design.

As regards pathogenesis, it was hypothesized that the lesions are linked to an alteration in the oxygen supply of ameloblasts with a consequent decrease of enamel mineralization [10]. In particular, every systemic physiological stress may influence the ameloblasts' activity before or at birth and in the first years of life [11].

Several prenatal (i.e., diabetes and hypocalcemia), perinatal (i.e., premature birth and prolonged delivery), and postnatal (i.e., antibiotics and nutrition problems) etiological factors were proposed; however, to date, there is no conclusive evidence on MIH etiology [12].

For this reason, further studies are needed in order to individuate the etiology of MIH.

Therefore, the purpose of the present study was to investigate the possible etiological factors potentially involved in the onset of MIH.

## 2. Materials and Methods

In the present study, 60 children (25 boys and 35 girls; average age: 9.8 ± 1.8 years) with MIH (test group) and 60 children (27 boys and 33 girls; average age: 10.1 ± 2 years) without MIH (control group) were included. Patients were selected at the unit of pediatric dentistry of the university hospital. The control group consisted of patients in general good health and without MIH, that were comparable to the test group for age and sex.

A written consent (signed by parents or legal guardians) to participate in the study was obtained for each child, and all procedures were conducted in accordance with the Declaration of Helsinki. Moreover, this study was approved by the Ethics Committee.

The inclusion criteria were as follows: age from 6 to 13 years, Caucasian, presence of at least one permanent molar with a MIH with or without the incisors involved (for the test group).

The exclusion criteria were as follows: the presence of hypoplastic lesions or hypomineralization, fluorosis, amelogenesis imperfecta, tetracycline stains, and history of dental trauma for the incisors.

The clinical dental examination was performed by the same operator, who is an expert in pediatric dentistry and who had received extensive training on clinical pictures of MIH lesions; each tooth affected by MIH was cleaned with a rotating brush, and tartar deposits were removed with ultrasound. Clinical examination of MIH was performed on wet teeth after cleaning. The criteria used to diagnose MIH were those indicated by Weerheijm et al., and for each tooth involved, the severity was assessed: mild (color change of the smooth surface without enamel defects), moderate (loss of enamel without dentine involvement), or severe degree (dentine involvement, atypical restorations, and teeth extracted because of severe lesions) [13].

All patients of the test group were reevaluated by a second operator in order to confirm the diagnosis and the score of MIH.

A questionnaire was distributed to parents in order to investigate the possible etiological factors of MIH which were divided into prenatal, perinatal, and postnatal parameters.

Each variable was linked to the child or parental history, especially the mothers.

### 2.1. Statistical Analysis

The chi-square test was used for the clinical parameters (severity and distribution).

Univariate and multivariate statistical analyses, based on the general linear model procedures, were used to investigate the relationship between MIH and etiological factors. The variables were included in multivariate analysis when they were found to be significant in statistical univariate analysis.

We calculated the odds ratios and 95% confidence intervals for each parameter included in the questionnaire, and the Wald test was used to test all standard hypotheses in the univariate and multivariate model; the level of significance was set at *p* < 0.05.

The statistical analysis was performed using the SPSS (Statistical Package for Social Sciences, Chicago, USA) 22.0 program.

## 3. Results

It was observed that 32 (53.3%) children of the test group exhibited both molar and incisor involvement, while 28 (46.7%) patients had only molars affected. No statistically significant difference was found between the two percentages (chi-square test *p*=0.47; relative risk 1.14; confidence interval 0.8–1.64).

The severity of MIH is reported in Table 1.

As regards molars, moderate lesions were significantly (*p* < 0.05) more frequent in comparison to both mild defects (chi-square test *p*=0.001 ; relative risk 1.48; confidence interval 1.22–1.81) and severe lesions (chi-square test *p*=0.001; relative risk 1.76; confidence interval 1.45–2.14). No significant difference was detected between mild molar defects and severe lesions frequencies (chi-square test *p*=0.07; relative risk 0.8; confidence interval 0.62–1.04).

As regards incisors, mild defects were significantly more frequent in comparison to moderate (chi-square test *p*=0.001; relative risk 3.12; confidence interval 2.16–4.51) and severe lesions (chi-square test *p*=0.001; relative risk 4.93; confidence interval 3.41–7.14).

Furthermore, moderate incisor lesions were significantly higher than severe lesions (chi-square test *p*=0.002; relative risk 1.93; confidence interval 1.54–2.42).

Table 2 provides the prevalence of prenatal, perinatal, and postnatal etiological variables associated with MIH in the two groups.

Univariate analysis showed a statistically significant correlation (*p* < 0.05) between MIH and antibiotics, infectious diseases, and respiratory and ear, nose, and throat (ENT) disorders.

However, with multivariate analysis, a statistical association (*p* < 0.05) was found only between MIH and antibiotics and ENT disorders (Table 3).

## 4. Discussion

MIH diagnosis might be difficult to outpoint, and a standardized protocol is necessary for the dentists and for the reports in epidemiological studies of MIH [14].

As regards the lesion distribution, a slight higher prevalence of both molar and incisor involvement was observed, while the percentage of children showing only molar involvement were slightly lower. The results in the literature are partially contradictory: Jasulaityte et al. found that 77.4% of schoolchildren exhibited MIH defects only in molars, while a lower percentage (22.6%) showed both molar and incisor enamel lesions [15].

However, in the Dutch National Epidemiological Survey of 2003, a higher percentage (57.1%) of both molar and incisor involvement was recorded [16].

Also in a study conducted on 360 Greek children aged 8–12 years, it was observed that a lower percentage of patients (28.4%) showed only molars affected, while more than 70% of the children exhibited both incisor and molar lesions [17].

The results of our study showed that mild defects were more frequent in molars. In the literature, it was observed by Buchgraber et al. [18] that demarcated enamel opacities were the most frequent lesions in Austrian children aged 6–12 years.

Jasulaityte et al. [16] found that children with MIH exhibited only demarcated opacities in high percentage (55.6%), while 20.6% of the patients showed at least one tooth with occlusal breakdown. These data are partially in agreement with the results of our study as we found a 21.5% of severe lesions in molars.

In our research, it was noticed that incisors showed milder enamel defects in comparison to molars, and this result was in line with a previous study, conducted on 277 children aged 8–12 years, showing that the severity of MIH defects was significantly more in molars compared to incisors [19].

Moreover, also in a recent study conducted on 154 Malaysian children aged 7–12 years, it was found that incisor involvement was less frequent than molar involvement (58%) and mild lesions exhibited a very high percentage (96.6%) [20].

Our results are also in agreement with those recorded by Lygidakis et al. [17] that observed a higher percentage (37.9%) of molars with moderate or severe lesions in comparison with incisors (4.9%); in both incisors and molars, mild defects were detected in highest prevalence (62.1% in molars and 95.1% in incisors).

In the present study, a positive association was observed between MIH and antibiotics (penicillin use) and ENT disorders even if, in the literature, this association is still uncertain.

Mulic et al. [3] examined 103 children with MIH and found that the use of penicillin due to adenoid infections in the first five years was associated with a higher prevalence of enamel lesions.

Furthermore, Laisi et al. [21] stated that an altered pattern of amelogenesis may interfere with the process of enamel mineralization and that the early use of amoxicillin is one of the main causative factors of MIH. However, these results should be interpreted with caution as it is not possible to determine if it is the antibiotic use or the disease or a combination of both that can lead to the enamel lesions.

In the literature, other possible etiological factors of MIH were also found; in fact, in a recent systematic review, a positive association was observed between maternal alcohol consumption, infantile fever, and ethnicity; however, as the authors stated, the validity of these results was impaired by poor study design and other methodological errors [9]. In the present study, no association between MIH and prenatal and perinatal variables was found, and these results are in accordance with those reported by Basak et al. [22].

A limitation of this study, in addition to the small number of patients included, is that, using a questionnaire, there is a lack of validity, as the respondent may be forgetful or not be thinking within the full context of the situation. Furthermore, the present study was limited only in our region, and the results of this study may not accurately reflect a larger international sample.

## 5. Conclusion

In the present study, similar percentages of only molar involvement and molar/incisor involvement were detected. Furthermore, it was observed that molars exhibited higher severity scores in comparison to incisors.

The results of this study show that, among the possible etiological factors of MIH, antibiotics and ear, nose, and throat diseases, during the first years of life, seem to be the predominant factors, while no positive association between prenatal/perinatal factors and MIH lesions was found.

## Figures and Tables

**Table 1:** Severity score.

	Mild	Moderate	Severe
Molars	55 (29.6%)	91 (48.9%)	40 (21.5%)
Incisors	75 (76.5%)	20 (20.4%)	3 (3.1%)

**Table 2:** Prenatal, perinatal, and postnatal variables (percentages) in the test group (with MIH) and in the controls (without MIH).

Possible etiological factors	Test group (%)	Control group (%)
Fluoride supplements in pregnancy	8	6
Gestational diabetes	27	13
Drug use in pregnancy	22	13
Full-term baby delivery	82	73
Smoking during pregnancy or breastfeeding	11	13
Natural vaginal birth	60	73
Childbirth complications	9	7
Breastfeeding	58	40
Allergies	24	13
Penicillin	84	27
Vitamin D	53	27
Infectious diseases	55	13
Ear, nose, and throat (ENT) disorders	60	7
Respiratory disorders	31	7
Physiological weaning for breastfeeding	75	87

**Table 3:** Univariate and multivariate analysis for etiological factors of MIH.

Variable	Univariate analysis			Multivariate analysis		
	*p*	OR	95% CI	*p*	OR	95% CI
Breastfeeding	0.236	0.487	0.148–1.602			
Allergies	0.373	0.476	0.093–2.443			
Antibiotics	0.001^∗^	0.067	0.017–0.272	0.019^∗^	0.138	0.026–0.717
Vitamin D	0.081	0.318	0.088–1.151			
Childbirth complications	0.788	0.732	0.075–7.113			
Gestational diabetes	0.301	0.423	0.083–2.157			
Drug use in pregnancy	0.461	0.538	0.104–2.793			
Fluoride supplements in pregnancy	0.623	0.571	0.061–5.323			
Smoking during pregnancy or breastfeeding	0.817	1.231	0.213–7.119			
Full-term baby delivery	0.459	0.595	0.150–2.354			
Infectious diseases	0.044^∗^	0.192	0.039–0.953	0.644	0.628	0.087–4.535
Ear, nose, and throat (ENT) disorders	0.005^∗^	0.048	0.006–0.395	0.038^∗^	0.093	0.010–0.880
Respiratory disorders	0.058^∗^	0.158	0.019–1.324	0.755	0.678	0.059–7.837
Natural vaginal birth	0.357	1.833	0.504–6.663			
Physiological weaning	0.373	2.103	0.409–10.80			

^∗^ *p* < 0.05

## Data Availability

The data sets generated during and/or analyzed during the current study are available from the corresponding author on reasonable request.
